# Exosome-mediated cellular crosstalk within the tumor microenvironment upon irradiation

**DOI:** 10.20892/j.issn.2095-3941.2020.0150

**Published:** 2021-02-15

**Authors:** Chuanshi He, Ling Li, Linlin Wang, Wanrong Meng, Yaying Hao, Guiquan Zhu

**Affiliations:** 1 Department of Stomatology, Sichuan Cancer Hospital, Sichuan Key Laboratory of Radiation Oncology, School of Medicine, University of Electronic Science and Technology of China; 2 State Key Laboratory of Oral Diseases, National Clinical Research Centre for Oral Diseases, Department of Head and Neck Oncology, West China Hospital of Stomatology, Sichuan University, Chengdu 610041, China

**Keywords:** Radiotherapy, tumor microenvironment, exosome, irradiation, extracellular vesicle

## Abstract

Radiotherapy is one of the most effective treatment methods for various solid tumors. Bidirectional signal transduction between cancer cells and stromal cells within the irradiated microenvironment is important in cancer development and treatment responsiveness. Exosomes, initially considered as “garbage bins” for unwanted from cells, are now understood to perform a variety of functions in interactions within the tumor microenvironment. Exosome-mediated regulation processes are rebuilt under the irradiation stimuli, because the exosome production, uptake, and contents are markedly modified by irradiation. In turn, irradiation-modified exosomes may modulate the cell response to irradiation through feedback regulation. Here, we review current knowledge and discuss the roles of exosome-mediated interactions between cells under radiotherapy conditions.

## Introduction

Radiotherapy (RT) is currently one of the most effective treatment modalities for various solid tumors^1–3^. Direct killing of tumor cells by RT is a commonly recognized anti-tumor mechanism^4^. In addition to tumor cells, numerous types of stromal cells are found in the tumor microenvironment, including fibroblasts, mesenchymal stem cells, lymphocytes, mononuclear phagocytes, polymorphonuclear phagocytes, and antigen-presenting cells. The radiation-induced bystander effect (RIBE) is defined as the biological response of non-targeted, non-irradiated cells after nearby cells are irradiated—a phenomenon thought to occur through cell-to-cell communication within the tumor microenvironment^5^. A previous study has shown that RIBE can injure non-irradiated cells. In one proposed mechanism, this process is facilitated by intercellular communication, through soluble factors released by irradiated cells and organs, as well as gap junction channels between irradiated cells^6^. However, the mechanisms through which RIBE occurs have not yet been fully elucidated.

Extracellular vesicles (EVs), which have gained increasing attention in the past decade, carry a variety of signaling molecules and provide a new avenue for facilitating cell-to-cell communication^7^. According to MISEV2018, EVs are the generic term for particles released naturally from cells^8^. EVs, including exosomes, microvesicles (MVs), and apoptotic bodies, are nano-sized membrane vesicles produced by most cell types^8,9^. A growing body of evidence indicates that EVs are important mediators of intercellular communication that play major roles in numerous physiologic and pathologic processes, including stem cell maintenance, tissue repair, immune modulation, and tumor growth^10–13^. Exosomes, a class of EVs, have attracted attention in cancer research because of their small size and the discovery that they function as molecular carriers that facilitate the delivery of information and signals between cells^14^. Because of these properties, exosomes have been implicated in the regulation of cancer sensitivity^15^ or resistance to radiation, as well as in the underlying mechanism of RIBE^16^.

In this review, we focus on the role of exosomes in radiotherapy by reviewing current knowledge and discussing the role of exosome-mediated interactions between cells during radiotherapy.

## Exosomes

The term “exosome” was first proposed in the 1980s^17^. Exosomes are nanoscale vectors vesicles with diameters of 30–100 nm that are secreted into the extracellular space through the fusion of multivesicular bodies with the plasma membranes of cells^18^. Exosomes show a typical cup-like morphology with negative staining, but they appear round when observed through transmission electron microscopy and cryo-electron microscopy^19,20^. Exosomes were initially considered to be simple molecular garbage bins that packaged and delivered waste components from cells^17^. However, in 1996, Raposo et al.^21^ revealed that exosomes secreted by B lymphocytes are able to elicit antigen-presenting effects and stimulate T lymphocyte activity. Since then, researchers have paid substantial attention to exosomes and their functions in intercellular communication.

### Biogenesis

The biogenesis of exosomes begins with the budding of cell membranes from multivesicular bodies (MVBs) to form intraluminal vesicles (ILVs); these MVBs are then transported to, and fuse with, the plasma membrane of the cell, thereby releasing exosomes^22^. Endosomal sorting complex required for transport (ESCRT) is a family of proteins that form successive complexes (ESCRT-0, -I, -II, and -III) at the membranes of MVBs, thereby regulating ILV formation and the targeting mechanisms for cargo^23^. In some cases, ESCRT-independent mechanisms, which use lipids, tetrameric proteins, or heat shock proteins, are instead used to form MVBs. Exosome secretion begins with the docking of the MVB to the cell membrane, in a process mediated by the RAB protein, followed by membrane fusion and release of the exosome into the extracellular space^24^; this fusion is facilitated by the soluble NSF-attachment protein receptor (SNARE) complex^25^.

The mechanisms that drive the mobilization of MVBs and their fusion with cell membranes include sophisticated regulatory processes involving a variety of molecules. The biogenesis of exosomes is therefore vulnerable to changes in the expression and function of these molecules.

### Components

The number of reports regarding the components of exosomes has increased over the past decade. To date, 769 proteins, 1116 lipids, 3408 mRNAs, and 2838 miRNAs have been identified according to ExoCarta, EVpedia, and Vesiclepedia^26–28^. The various protein components of exosomes include membrane transport and fusion proteins (e.g., Rab GTPases, annexin, and flotillin), heat shock proteins (hsc70 and 90), integrins, and 4 transmembrane proteins (CD63, CD9, CD81, and CD82)^29,30^. Several proteins that are highly associated with exosomes have been defined as exosomal marker proteins (e.g., Alix, flotillin, TSG101, and CD63)^31^. Recent sequencing analysis results have shown that exosomes contain a variety of mRNAs, miRNAs, and other small non-coding RNA species, such as RNA transcripts that are repeated with protein coding regions, structural RNA, tRNA fragments, vault RNA, Y RNA, and small interfering RNA^32^. Furthermore, exosomes are enriched with long acyl chains of saturated fats, which are frequently found in lipid raft components, such as cholesterol, sphingomyelin, ceramides, and glycerophospholipids^33^.

Exosomes that originate from different cells contain diverse contents that exert different functions. For example, tumor-derived exosomes that contain exosomal annexin II (exo-Anx II) exert an angiogenic effect in addition to playing an important role in the metastasis of breast cancer^34^. Furthermore, plasma-derived exosomes are capable of transmitting endogenous protection signals to the myocardium through the HSP70/TLR4 axis^35^. Researchers have elucidated the functions of many nucleic acids carried by exosomes, particularly miRNAs, in a variety of physiologic and pathologic scenarios. However, additional research is required to further investigate the characteristics and functions of molecules carried by exosomes under specific conditions.

## Radiotherapy regulation of exosome production

The secretion ability and the quantity of secreted exosomes vary across cell types and involve complex mechanisms that govern exosome function and release; these mechanisms have substantial heterogeneity among cell types. Several molecular mechanisms involved in the secretion of exosomes have been explored and confirmed. The MAPK^36^ and p53 pathways^37^, for instance, have been found to be involved in the regulation of exosome secretion. Lespagnol et al.^38^ have identified a target gene of p53—tumor suppressor-activated pathway 6 (TSAP6), also known as Steap3—that is involved in the production and secretion of exosomes. In future studies, exploration of the secretory mechanism of exosomes and the elements involved in this process are needed, because certain types of exosomes could potentially serve as cancer biomarkers, treatment targets, or drug delivery systems.

### Exosome secretion

Previous studies have reported that radiation enhances the release of exosomes from both malignant glioma cells and normal astrocytes^39^. Exosomes derived from irradiated glioma cells enhance the migration of recipient cells. Furthermore, their molecular profiles have revealed an abundance of molecules associated with signaling pathways important for cell migration^39^. NanoSight measurements have also shown a greater number of exosomes recovered from irradiated head and neck cancer cells than from non-irradiated cells 24 h after irradiation^40^. Lehmann et al.^41^ have reported that radiation promotes the release of prostate cancer-derived exosomes into the microenvironment, thereby affecting tumor cell senescence. However, although these reports describe the effects of radiation on exosome secretion, they do not illustrate the mechanisms responsible for these effects. Recently, Bagheri et al.^42^ have demonstrated that the secretion of human endothelial-derived exosomes is enhanced when cells are exposed to low-level laser irradiation at high power; this increase in secretion is mediated through the stimulation of transcription factors associated with Wnt signaling and autophagy. Further investigations are needed to reveal the detailed mechanisms by which radiation alters the secretion of exosomes. Discovering an effective shortcut for promoting exosome secretion will make the industrial production of exosomes a reality and unlock the potential for applications in clinical practice.

### Exosome uptake

Exosomes, after their release into the extracellular environment, are taken up by recipient cells, both neighboring and distant, where they perform their regulatory functions. Exosomes interact with their recipients through the adhesion molecules and ligands located on the surfaces of the bilayer membrane^43,44^; they are then internalized, and their contents are released into the cell, in which they regulate protein activity and gene expression; thus, exosomes play major roles in modifying the physiologic and pathologic processes of their recipients^45^. Because of their high biocompatibility and stability, and limited immunogenicity, exosomes are considered nano-sized targeting tools for molecular targeted therapies against cancer^46,47^. Zhu et al.^48^ have demonstrated that exosomes derived from natural killer (NK) cells can target malignant glioma cells in a mouse model. However, exosome-dependent targeted therapy is far from being sufficiently developed for clinical use; further investigation is needed to explore the mechanisms involved in the uptake of exosomes by recipient cells.

Previous studies have reported that the cellular uptake of exosomes is affected by radiation exposure. Hazawa et al.^49^ have found that irradiation increases the cellular uptake of exosomes through the formation of CD29/CD81 complexes. The potential enhancement of exosome-based targeted drug delivery by irradiation could pave the way for the development of combined radio- and exosome-based targeted therapies for cancer.

## Radiotherapeutic exosome biomarkers

A growing body of evidence suggests that tumor-derived exosomes with miRNAs can potentially be used as biomarkers for early diagnosis of cancer^50^. Exosomes in the urine have been extensively investigated for their possible use in diagnosis and prognostication of prostate cancer^51^. Furthermore, the use of serum-derived exosomes as biomarkers has also been demonstrated in gliomas^52^, liver cancers^53^, endometrial cancers^54^, gastrointestinal cancers^55^, and other types of cancers.

The components carried by exosomes are affected by irradiation. Exosomal cargos can theoretically predict the effects of radiotherapy for different groups of patients and consequently be used as biomarkers. For example, the expression of miR-663a, as detected in exosomes released by hypoxic colorectal cancer cells, has been confirmed to be associated with resistance to radiotherapy elicited by the hypoxic tumor microenvironment^56^. Moreover, An et al.^57^ have performed a proteomic analysis of serum exosomes from patients with locally advanced pancreatic cancer at different time points during chemoradiotherapy and detected specific changes in the expression of 8 proteins associated with metastasis and resistance to therapy^57^. Furthermore, the authors have confirmed that the exosomes extracted from these patients promote the metastasis of tumor cells^57^. These results provide new perspectives for exploring pancreatic cancer metastasis and resistance to radiotherapy. However, the authors did not explore the mechanisms by which the exosomal contents could hypothetically regulate cancer metastasis and radiation resistance. Luo et al.^15^ have demonstrated that exosomal miR-339-5p modulates the radiosensitivity of locally advanced esophageal squamous cell carcinoma by downregulating the expression of the target protein Cdc25A. This study provides a basis for developing a novel method of identifying biomarkers specific for esophageal squamous cell carcinoma. Kulkarni et al.^58^ have investigated the proteomes of urine and serum exosomes from patients undergoing radiotherapy and have found that urine derived exosomes can be used to detect radiation damage in the liver and gastrointestinal and genitourinary systems. Serum exosomes have a positive effect on radiation-related vascular injury and the acute inflammatory response. Radiotherapy increases the quantity of serum exosomes and upregulates miRNA expression in patients with prostate cancer^59^. Yu et al.^60^ have confirmed that several specific, highly expressed miRNAs may be used to predict the sensitivity of cells to carbon ion radiotherapy (CIRT) treatment. MiR-493-5p, miR-323a-3p, miR-411-5p, miR-494-3p, miR-379-5p, miR-654-3p, miR-409-3p, miR-543, and miR-200c-3p have been detected in serum exosomes of patients with prostate cancer who underwent CIRT^60^. Additionally, the expression levels of miR-654-3p and miR-379-5p in exosomes after CIRT have also been found to be associated with treatment efficacy^60^. These results confirm that because the expression of miRNAs in serum exosomes is altered by radiation, these miRNAs might serve as biomarkers for monitoring the response to therapy. However, how these modified miRNAs regulate radio-sensitivity has not yet been verified.

Collectively, these reports confirm that radiation can alter the information transmitted by exosomes, which subsequently modifies the phenotype of recipient cells. The modification of exosomes by irradiation sheds light on the potential use of exosomes as specific biomarkers during radiotherapy for a variety of cancers. However, current biomarker studies have devoted much of their attention to only exosomal RNAs (**Table 1**); therefore, more studies are needed to investigate other exosomal cargos, such as proteins. Furthermore, the mechanisms underlying the changes in specific molecules after radiotherapy remain unclear.

**Table 1:** Potential function of exosomal RNAs in radiotherapy

Exosomal RNAs	Cancer	Potential function	Reference
AHIF	Glioblastoma	AHIF is upregulated and associated with radiation resistance	^61^
miR-1246	Breast cancer	Irradiation induces an increase in miR-1246 in TDEs, thus inhibiting the proliferation of non-irradiated cells	^62^
miR-23a	Nasopharyngeal carcinoma	TDEs deliver miR-23a to HUVECs, thus promoting tumor angiogenesis by targeting TSGA10	^63^
miR-23a	Lung cancer	Irradiated TDEs enhance the angiogenesis of HUVECs *via* the miR-23a/PTEN pathway	^64^
miR-339-5p	Esophageal cancer	Exosome-derived miR-339-5p mediates radiosensitivity by targeting Cdc25A	^15^

AHIF, antisense transcript of hypoxia-inducible factor; TDEs, tumor-derived exosomes; HUVECs, human umbilical vein endothelial cells.

## Tumor-derived exosomes mediate crosstalk between cancer cells in radiotherapy

Tumor-derived exosomes (TDEs) have been suggested to alter the physiologic and pathologic processes of cells and promote tumor cell growth^65^ and metastasis^66^ by delivering specific molecules to their recipients. Several experiments have demonstrated that radiation has significant effects on exosomal cargos^15^. Yentrapalli et al.^67^ have reported that radiation exposure changes the protein and miRNA components of purified exosomes in plasma. Proteomic analysis has revealed significant changes in 9 proteins, and immunoblotting has revealed downregulation of the expression levels of afamin and serpine peptidase F1. Furthermore, the expression levels of miR-204-5p, miR-92a-3p, and miR-31-5p have been found to be markedly enhanced^67^. Therefore, exosomes derived from irradiated tumor cells may alter the behavior of both adjacent and distant cells.

### Squamous cell carcinoma of the head and neck

Radiotherapy is a conventional treatment for squamous cell carcinomas of the head and neck. Jelonek et al.^68^ have used liquid chromatography-tandem mass spectrometry to demonstrate that radiation alters the protein content of exosomes derived from human head and neck squamous cell carcinomas. Their results showed that 236 proteins were expressed specifically in irradiated exosomes and that 69 of these proteins disappeared after irradiation was discontinued^68^. The presence of specific protein components in irradiated cells is often associated with the processes of transcription, translation, protein turnover, cellular division, and cellular signaling^68^. A similar conclusion has also been reached by Abramowicz, who demonstrated that irradiation affected the expression levels of 472 proteins—425 upregulated and 47 downregulated—in head and neck cancer cells^69^. The effects of radiotherapy on the molecular components carried by TDEs are clear. Because the effects of radiation are observed in such many molecular components, significant changes in numerous biological functions are expected to occur as well, thus highlighting the importance of exploring the molecular mechanisms involved in this phenomenon. Mutschelknaus et al.^40^ have found that irradiation can enhance the uptake of exosomes by affected cells. Functional analysis has revealed that exosomes from both non-irradiated and irradiated donor cells increases the proliferation of non-irradiated recipient cells and promotes the survival of irradiated recipient cells^40^. However, this enhancement of cell survival is more pronounced when exosomes isolated from irradiated, rather than non-irradiated, donor cells are used^40^. Furthermore, an increase in the repair of DNA double-strand breaks has been suggested to explain the enhanced survival of cells after receiving exosomes from irradiated cells^40^. Moreover, exosomes derived from irradiated head and neck squamous cell carcinoma cells have been found to promote metastasis in recipient cells^70^. The protein components of exosomes might potentially be regulated by radiation and subsequently stimulate the AKT signaling pathway and enhance cell metastasis^70^. These results should help improve the efficacy of radiotherapy for treating squamous cell carcinomas of the head and neck. However, the mechanisms underlying this phenomenon, including the proteins, non-coding RNAs, and signaling pathways involved, have not yet been elucidated and require further investigation.

### Glioblastoma multiforme

Glioblastoma multiforme (GBM) has the highest mortality rate among patients with brain tumors, with a 5-year survival rate of 4%–5%; radiotherapy is an important part of its treatment^71,72^. Antisense transcripts of hypoxia-inducible factor-1α (AHIF), a long non-coding RNA, are upregulated in GBM and are associated with radiation resistance^61^. Dai et al.^73^ have demonstrated that the expression of AHIF is upregulated in response to irradiation. Furthermore, exosomes derived from AHIFknockdown GBM cells inhibit viability and invasion, as well as decrease the resistance to radiotherapy in GBM. In contrast, exosomes derived from AHIFoverexpressing GBM cells promote these processes^73^. These results indicate that AHIF-carrying, radiation-modified TDEs may function as regulators of radio-sensitivity in GBM and thus are potential therapeutic targets. Furthermore, Arscott et al.^39^ have demonstrated that the molecular changes present in exosomes derived from irradiated cells augment signals involved in cell migration in recipient cells. The authors have likewise demonstrated that exosomes released by irradiated GBM cells enhance the migration of recipient tumor cells and promote the expression of connective tissue growth factor (CTGF) mRNA and insulin-like growth factor binding protein 2 (IGFBP2)^39^. Furthermore, exosomes secreted by irradiated cells enhance the activity of migration-associated molecules in recipient cells, such as neurotrophic tyrosine kinase receptor type 1, focal adhesion kinase, paxillin, and the proto-oncogene tyrosine-protein kinase Src^39^. These reports suggest that irradiation modifies the production of exosomes and their molecular composition, thereby promoting tumor progression. Elucidating the molecular mechanisms underlying these exosome changes during radiotherapy may provide new therapeutic options for use against radiation resistant cells. On this basis, exosomes have the potential to become an integrated platform for individualized radiotherapy and prognosis assessment in GBM.

### Non-small cell lung cancer

Lung cancer has the highest morbidity and mortality rates worldwide among all cancers. Non-small cell lung cancer (NSCLC), the most common subtype, exhibits a very low 5-year survival rate. Approximately 75% of patients are diagnosed in moderate to advanced stages of the disease and often require radiotherapy^74^. Radiation can alter the cargo carried by NSCLC-derived exosomes, such as by decreasing the levels of miR-29a-3p and miR-150-5p, thus allowing improvements in the effectiveness of radiotherapy for patients with this disease^75^. Wu et al.^76^ have confirmed that irradiation enhances the activity of anaplastic lymphoma kinase (ALK) in exosomes secreted by NSCLC cells. These exosomes activate the AKT, STAT3, and ERK pathways in recipient cells, thereby decreasing the sensitivity of tumor cells to ceritinib and ultimately promoting tumor growth^76^. Radiation-induced ALK activity in NSCLC-derived exosomes may be a key factor in promoting tumor progression and resistance to ALK inhibitors. These results provide a new reference for the adjuvant effects of ALK inhibitors during radiation therapy for NSCLC. The effectiveness of treatment is always confounded by resistance to radiotherapy in NSCLC patients. However, current results are not sufficient to quantify exosome components. Attention must be paid to the exploration of mechanisms, which have a decisive role in improving the radiotherapy effects in NSCLC.

**Figure 1:**
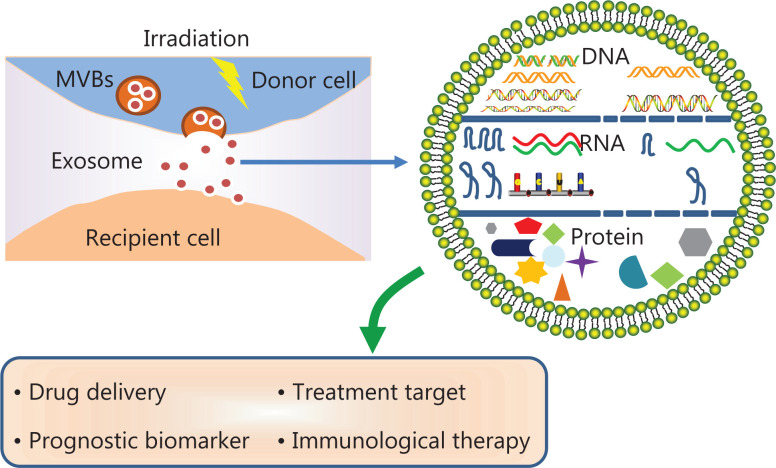
Schematic representation of the effects of radiotherapy on exosome production and contents.

A growing body of evidence indicates that radiation alters the cargo carried by TDEs. Here, we present a schematic diagram of the effects of radiation on exosome production and composition (**Figure 1**). However, most of these reports have focused only on pre- and post-radiation changes in exosomal proteins and miRNAs, whereas the mechanisms involved in these changes and their effects on biological functions have not been elucidated and require further investigation.

## Tumor derived exosomes regulate non-targeting effects in radiotherapy

In addition to tumor cells, many types of stromal cells are present in the tumor microenvironment. Lundholm et al.^77^ have demonstrated that prostate cancer-derived exosomes downregulate the expression of NKG2D in NK and CD8+ T cells, thus decreasing NKG2D-mediated cytotoxicity and promoting immune suppression and tumor escape. TDEs inevitably affect surrounding normal cells. Tumor-derived exosomes can also be taken up by various stromal cells in the tumor microenvironment located as far as the edge of the irradiated area. In early 2012, Al-Mayah et al.^78^ reported that RNAs in exosomes are involved in mediating the NTEs of radiation therapy. Therefore, TDEs act not only on themselves, but also on stromal cells and non-irradiated cancer cells; this phenomenon should play a crucial role in the modification of the tumor microenvironment during radiotherapy.

### Tumor derived exosome-regulation of the biology of non-irradiated cancer cells

Recently, the non-targeting effects (NTEs) of ionizing radiation, which include genomic instability and bystander effects, have received much attention in the field of radiation therapy. In 2015, exosomes were revealed to be associated with prolonged promotion of the NTEs of irradiation^6^. The RNA and protein molecules carried by exosomes synergistically initiate NTE, spread these effects to naive cells, and perpetuate genomic instability in affected cells^6^. In addition, RNase treatment and thermal denaturation (by boiling) of exosomes derived from mammary epithelial cells protects telomerase, and consequently telomere length, from bystander effects. These results suggest that exosomal RNA and protein from irradiated mammary epithelial cells contribute to the production of bystander effects^79^. Additionally, exosomes extracted from ultraviolet biophoton-irradiated colon carcinoma cells, but not non-irradiated cells, have been found to produce bystander effects in reporter cells^80^. Treating exosomes with RNase before their incubation with reporter cells effectively abolishes bystander effects, thus suggesting that exosomal RNA plays an important role in mediating the bystander response^80^. These results demonstrate that radiation-modified exosomal RNA mediates the NTEs of irradiation. However, the details regarding the mechanisms and specific exosomal RNAs involved in this phenomenon remain unclear. Mo et al.^62^ have demonstrated that irradiation induces an increase in miR-1246 levels in TDEs, thereby inhibiting the proliferation of non-irradiated cells. MiR-1246-rich TDEs increase the yield of the DNA double-strand break biomarkers 53BP1 foci, comet tails, and micronuclei in non-irradiated cells while decreasing the efficiency of non-homologous end joining. The authors have concluded that TDEs containing miR-1246 act as transfer messengers and contribute to radiation-induced bystander DNA damage by directly repressing the LIG4 gene^62^. These results suggest that exosomes may regulate radiation-induced genomic instability and bystander effects. Current results collectively reveal that TDEs, through radiation, have a two-way role in non-irradiated cancer cells, by not only promoting the proliferation of cancer cells but also killing them. The mechanisms underlying this process, however, remain poorly understood and require further investigation.

### Radiation-modified TDEs regulate dendritic cell biology

Dendritic cells (DCs) are the most powerful antigen-presenting cells in the body and are found abundantly in the tumor microenvironment. A pioneer study performed by Diamond et al.^81^ has shown that exosomes produced by irradiated mouse breast cancer cells (RT-TDEs) transfer dsDNA to DCs, thus increasing the production of costimulatory molecules and the STING-dependent activation of IFN-I. An *in vivo* study has demonstrated that irradiated TDEs elicit tumor-specific CD8+ T-cell responses and protect mice from tumor development significantly better than non-irradiated TDEs^81^. Overall, these results had indicated that TDEs act as a platform for transfer of dsDNA from irradiated cancer cells to DCs, thereby stimulating the production of IFN; this discovery may indicate potential benefits of combining RT with immunotherapy^81^. Another study performed by Hurwitz et al.^82^ has demonstrated that TDEs from irradiated human prostate cancer cells containing Hsp72 potentially stimulate the production of pro-inflammatory cytokines and certain co-stimulatory molecules in human DCs. These results highlight the roles of TDEs in radiation-induced DC-dependent immune responses and shed light on the potential applications of using immunotherapy concurrently with radiotherapy.

### Irradiation-modified TDEs regulate endothelial cell biology

Angiogenesis involves the sophisticated orchestration of endothelial cell activities, such as proliferation, migration, invasion, adhesion, and differentiation, under physiologic or pathologic conditions^83^. The presence of pathologic angiogenesis is necessary for tumor progression, because it provides oxygen, nutrients, and conduits for metastasis^84^. Exosomes derived from local nasopharyngeal carcinoma cells have been found to deliver miR-23a to human umbilical vein endothelial cells (HUVECs) and subsequently promote tumor angiogenesis by targeting TSGA10^63^.* In vitro* studies have demonstrated that pancreatic cancer-derived exosomes promote angiogenic activity through dynein-dependent endocytosis of endothelial cells^85^. Likewise, exosomes derived from colorectal cancer cells activate adjacent vascular endothelium by delivering microRNA200 to these cells^86^.

Upon irradiation, exosomes derived from gastric cancer cells promote the proliferation, migration, and invasion of HUVECs, but these responses are impaired by the VEGFR-2 inhibitor apatinib^87^. Additionally, exosomes derived from irradiated lung cancer cells enhance the angiogenesis of HUVECs by activating the miR-23a/PTEN pathway^64^. These results suggest that TDEs mediate a pro-angiogenic feature of irradiation and that patients may benefit from a combination of radiotherapy and administration of anti-angiogenic drugs.

## Stromal cell-derived exosomes in radiotherapy

As mentioned above, the crosstalk between tumor cells and different types of stromal cells is bidirectional and involves intricate mechanisms. We previously discussed the influence of tumor cells on stromal cells and other tumor cells *via* TDEs. Given that exosomes are secreted by nearly all cell types, exosomes derived from stromal cells could theoretically influence cancer cells and other types of stromal cells in the context of radiation exposure^88,89^; this concept will be discussed further below.

### Mesenchymal stem cell-derived exosomes

Exosomes from mesenchymal stem cells (MSCs) play functional roles in regulating radio-sensitivity. De Araujo Farias et al.^90^ have demonstrated that exosomes derived from MSCs improve the killing effect of radiotherapy on melanoma cells and control their potential for metastasis. Concomitant and adjuvant use of RT and MSC-derived exosomes, compared with radiotherapy alone, increases the survival time of mice by 22.5% and decreases the number of metastatic lesions by 60%^90^. Exosomes from mesenchymal stem cells also reverse tissue damage caused by irradiation. Hence, these results provide evidence the feasibility of using a combination of radiotherapy and administration of MSC-derived exosomes in treating cancer.

Radiation-induced lung injury is a common complication of radiotherapy that limits the effective therapeutic dose administered to tumors. Increasing evidence suggests a role of MSCs in modulating the inflammatory and immune responses in this complication, as well as in promoting the repair and regeneration of damaged lung tissue *via* exosomes^91^. MSCs have also been shown to reverse radiation-induced damage of bone marrow stem cells (BM-MSCs). Furthermore, exosomes secreted by mouse and human MSCs decrease DNA damage and apoptosis while promoting growth and proliferation in mouse BM-MSCs^92^. Zuo et al.^93^ have shown that BM-MSC-derived exosomes attenuate radiation-induced bone marrow damage in a rat model. The combination of radiotherapy and the administration BM-MSC-derived exosomes is more effective in reducing oxidative stress, promoting DNA damage repair, reducing cell proliferation inhibition, and attenuating cell senescence-associated protein expression than radiotherapy alone^93^. After irradiation, BM-MSC-derived exosomes promote the expression of β-catenin and restore the balance between adipogenic and osteogenic differentiation^93^. Gingival MSC-derived exosomes have also been shown to repair damage to taste buds caused by radiotherapy for oral cancer^94^.

Collectively, these studies demonstrate that exosomes derived from MSCs have great potential in preventing and repairing radiation damage, thus providing a basis for possible applications in clinical settings.

### Immune cell-derived exosomes

The effects of radiation therapy on the immune system are now attracting attention from clinicians and scientists. Many immune cells are involved in radioimmunotherapy, such as T cells, B cells, mononuclear macrophages, NK cells, and some antigen-presenting cells. Exosomes derived from tumor peptide-pulsed DCs have been found to prime specific cytotoxic T lymphocytes and suppress the growth of tumors in a T cell-dependent manner^95^. Indeed, because the uptake and presentation of antigens by DCs are influenced by irradiation^96^, DC functions in general might potentially also be influenced by irradiation. Schnitzer et al.^97^ have provided more evidence supporting this hypothesis by demonstrating that exosomes derived from irradiated antigen-loaded DCs can serve as effective vaccines and establish protective immunity. Furthermore, upon irradiation, T cell-derived exosomes inhibit the proliferation, but promote the metastasis, of esophageal squamous cell carcinoma cells in a dose- and time-dependent manner^98^. These results suggest that irradiation may alter exosomes derived from immune cells and contribute to the appearance of radiation-related effects in cells *via* a feedback mechanism. However, limited data are available on immune cell-derived exosomes and their roles in radiation biology; this is an interesting topic that warrants further investigation.

### Fibroblast-derived exosomes

Fibroblasts are one of the most abundant types of stromal cells found in the tumor microenvironment. Activation of cancer-associated fibroblasts promotes angiogenesis and the invasiveness, stemness, and chemoresistance of cancer cells, as well as the recruitment of immune cells, in the tumor microenvironment^99^. Boelens et al.^100^ have found that crosstalk between breast cancer cells and fibroblasts is orchestrated by RNA strands found inside fibroblast-derived exosomes; this crosstalk increases radiation resistance by breast cancer cells through the STAT1 and NOTCH3 signaling pathways^100^.

Currently, limited data are available on the mechanisms by which radiation mediates the secretion of stromal cell-derived exosomes, the contents of these exosomes, and the functions of these contents in cancer cell biology. Because exosomes derived from T lymphocytes^101^, DCs^102^, and NK cells^103^ exhibit functions and characteristics of their parent cells, they are now considered potential candidates for use in cancer therapy. Whether the distribution and uptake of these exosomes by cancer cells would be modified in an irradiated tumor microenvironment remains largely unknown; determining the answer to this question is a critical step in the development of exosome-based cancer therapies. Furthermore, whether irradiation affects the production and contents of stromal cell-derived exosomes has not been fully investigated. Because stromal cell-derived exosomes are an important component of bidirectional signal transfer between cancer cells and stromal cells within the tumor microenvironment following radiation exposure, further investigation of the effects of irradiation on the function of these exosomes should prove interesting.

## Perspectives

Both cancer cells and stromal cells are influenced by the physical and chemical characteristics of the tumor microenvironment, and crosstalk between these cells may also dynamically reshape the microenvironment through a feedback mechanism. Exosomes, which have been discovered to have functions in signal communication, have, in the past decade, advanced to the forefront of cancer research. In the tumor microenvironment, the production of exosomes, as well as exosomal proteins and nucleic acids, may be influenced by irradiation; therefore, exosomes can potentially be used as non-invasive diagnostic markers for radio-sensitivity or radio-resistance. Indeed, the use of exosomes as a platform for minimally invasive circulating biomarkers has gained much attention and is currently under intensive investigation.

**Figure 2:**
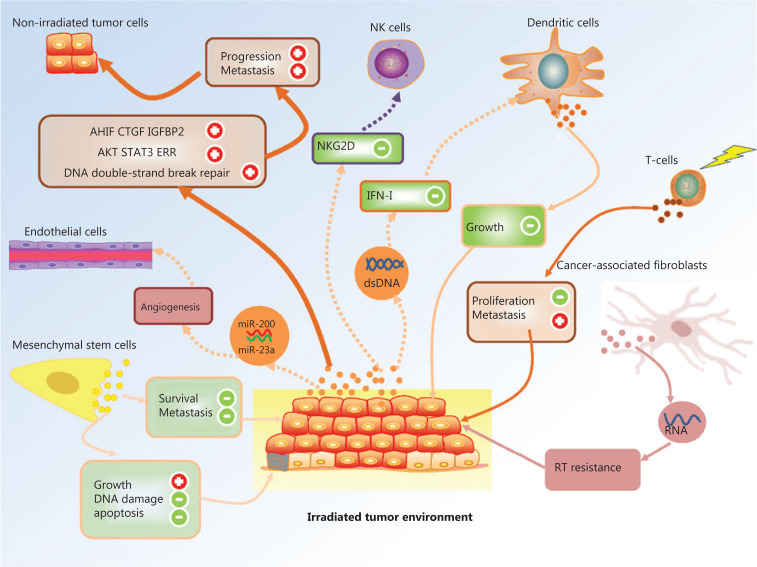
Schematic representation of the exosome mediated crosstalk between a variety of cell types within the irradiated tumor microenvironment.

Exosomes mediate a broad range of bidirectional signal transduction between various cell types, such as in cancer cell-to-cancer cell, cancer cell-to-stromal cell, and stromal cell-to-stromal cell communication, within the tumor microenvironment (**Figure 2**). They also play essential roles in regulating tumor angiogenesis, invasiveness, proliferation, chemotherapy resistance, evasion of the immune system, metabolism, and stemness. The regulation of these processes is radically modified by irradiation due to changes in the production and uptake of exosomes and their contents. Exosomes modified by irradiation may regulate cellular responses to irradiation through a feedback mechanism. Recently, the non-targeted effects of ionizing radiation, which include induction of genomic instability, bystander effects, and abscopal effects, have received much attention in the field of radiation therapy. However, the mechanisms underlying these non-targeted effects remain poorly understood. The growing knowledge of how exosomes mediate cell-to-cell communication provides clues for understanding the mechanisms behind non-targeted effects of irradiation.

Exosomes not only offer a new perspective in elucidating the mechanisms involved in radiotherapy resistance and the occurrence of non-targeted effects, but also represent a novel strategy in cancer therapy. Given that exosomes derived from T cells, DCs, and NK cells exhibit both anti-tumor effects and immunoregulatory functions, the potential use of exosomes as immunotherapy agents or as drug delivery platforms has received considerable scientific interest. In fact, the production, infiltration, distribution, and incorporation of stromal cell-derived exosomes under irradiation are important in exosome-based therapeutics but are far from being well-elucidated. Gaining a more comprehensive understanding of exosomes, their molecular cargos, and stromal origins, as well as their function in the regulation of radio-sensitivity and possible use in combination therapies, is both challenging and fascinating, because they should offer avenues to improve the evaluation and treatment of tumors in the future.
